# Alternative Preparation of Improved NiMo-Alumina Deoxygenation Catalysts

**DOI:** 10.3389/fchem.2020.00216

**Published:** 2020-04-07

**Authors:** Peter Priecel, David Kubička, Armando Vázquez-Zavala, José Antonio de los Reyes, Miroslav Pouzar, Libor Čapek

**Affiliations:** ^1^Department of Physical Chemistry, Faculty of Chemical Technology, University of Pardubice, Pardubice, Czechia; ^2^Unipetrol Centre for Research and Education, Litvínov, Czechia; ^3^Department of Petroleum Technology and Alternative Fuels, University of Chemistry and Technology Prague, Prague, Czechia; ^4^Departamento de Ingeniería de Procesos e Hidraulica, Universidad Autónoma Metropolitana-Iztapalapa, Mexico City, Mexico

**Keywords:** deoxygenation, NiMo-alumina, ethanol, preparation, impregnation, hydrotreating

## Abstract

This investigation deals with NiMo-alumina hydrotreating catalysts effective in the deoxygenation of rapeseed oil. The main goal was to compare catalyst structure and their deoxygenation performance and to link these parameters to reveal important structural information regarding the catalyst's intended use. Catalysts were prepared from different precursors (nickel acetate tetrahydrate/molybdenyl acetylacetonate in ethanol and water vs. nickel nitrate hexahydrate/ammonium heptamolybdate tetrahydrate in water), which resulted in their contrasting structural arrangement. These changes were characterized by elemental composition determination, UV-Vis diffuse reflectance spectroscopy, temperature programmed reduction by hydrogen, nitrogen physisorption at 77 K, scanning and transmission electron microscopies, and deoxygenation of rapeseed oil. The critical aspect of high oxygen elimination was a homogeneous dispersion of NiO and MoO_3_ phases on the support. It subsequently led to the effective transformation of the oxide form of a catalyst to its active sulfide form well-dispersed on the support. On the other hand, the formation of bulk MoO_3_ resulted in the separate bulk phase and lower extent of sulfidation.

## Introduction

Deoxygenation of vegetable oils or fats is an alternative process for obtaining renewable diesel-like fuel with a high cetane number (Bianchi, 2009). It has a disadvantage of high hydrogen consumption; however, it yields exclusively straight-chain alkanes and propane as the main products (Huber et al., 2007; Kubičková and Kubička, 2010). Hydrogen consumption can be decreased by using noble-metal-based catalysts (Kubičková et al., 2005; Murzin et al., 2009; Boda et al., 2010; Kubičková and Kubička, 2010) or by fine-tuning the parameters of conventional sulfide (Okamoto et al., 2002; Kubička and Kaluža, 2010; Bui et al., 2011) or a different phase, such as carbide, nitride, or phosphide (Ledoux et al., 1996; Diaz et al., 2003; Furimsky, 2003; Oyama, 2003; Oyama et al., 2009).

Generally, NiMo-based hydrotreating catalysts are prepared from ammonium heptamolybdate and nickel nitrate or oxides (Griboval et al., 1998; Breysse et al., 2008), eventually used with a complexing agent (Hiroshima et al., 1997; Shimizu et al., 1998; Sun et al., 2003; Escobar et al., 2008), e.g., citric acid (Pessimisis, 1984; Kubota et al., 2010; Pashigreva et al., 2010) or EDTA (Sundaramurthy et al., 2005; Lélias et al., 2010; Al-Dalama and Stanislaus, 2011), to improve the properties of the catalyst. Kang et al. (2019) recently highlighted reasons such as improvement in MoS_2_ active phase dispersion and stability against deactivation in hydroconversion of heavy oil.

Multiple groups focused on the synthesis using alternative precursors, such as nickel acetylacetonate (Kaluža and Gulková, 2016, Singh et al., 2016), molybdenyl acetylacetonate (Sakanishi et al., 2000; Farag, 2002; Kouzu et al., 2004; Kaluža and Gulková, 2016; Kang et al., 2019), or Mo(CO)_6_ (Singh et al., 2016) as opposed to classical nickel nitrate and molybdate. Kang et al. (2019) only recently reviewed various Mo-based precursors for the synthesis of catalysts for hydroconversion of heavy oil. They compared them with respect to the process important to the formation of the resulting catalyst, such as dispersibility or thermal properties of the precursor and sulfidation degree, particle size and slabs stacking of the final MoS_2_ phase. Within the review, the authors highlighted for example the study of Farag (2002) who confirmed the use of molybdenyl acetylacetonate results in the higher degree of sulfidation and formation of amorphous MoS_2_ phase (compared to heptamolybdate) and that the sulfidation of MoO_2_(acac)_2_ starts at a lower temperature.

Within similar precursors for NiMo catalysts, Kaluža and Gulková (2016) used Ni(acac)_2_ and MoO_2_(acac)_2_ in toluene and compared them to aqueous nickel nitrate and heptamolybdate to prepare ZrO_2_-based catalysts for hydrodesulfurization (HDS) of 1-benzothiophene and hydrogenation (HYD) of 1-methyl-cyclohex-1-ene. They found that organometallic precursors gave catalysts of 13% higher HDS activity and lower HYD/DDS (Direct DeSulfurization pathway) (at 50% conversion). Similarly, Sakanishi et al. (1995) screened carbon-based NiMo catalysts in hydrogenation of 1-methylnaphthalene and found 31% increase in HYD activity of catalyst prepared from methanol solution of Ni(OAc)_2_ and MoO_2_(acac)_2_ when compared to nickel nitrate and heptamolybdate (H_2_O/MeOH = 9/1).

We have previously reported the performance and structure of NiMo-alumina catalysts prepared by impregnation from nickel acetate tetrahydrate and molybdenyl acetylacetonate in ethanol solution and showed that alternative precursor could provide highly active and selective deoxygenation catalysts (Priecel et al., 2011a,b). The motivation for this study was to identify and describe key properties of NiMo-alumina catalysts with respect to their efficiency in the deoxygenation reaction, and to confirm if the precursors used here give a better catalyst with a higher population of active sites. For the more common NiMo-alumina preparation method using nickel nitrate hexahydrate and ammonium heptamolybdate tetrahydrate in water, we have chosen an alternative catalyst preparation method using nickel acetate tetrahydrate and molybdenyl acetylacetonate in ethanol/water. To the best of our knowledge, these two precursors were previously not used together to synthesize NiMo-alumina catalysts and used them for deoxygenation reaction.

## Materials and Methods

γ-Alumina, supplied by Euro Support Manufacturing Czechia (*S*_BET_ = 296 m^2^ g^−1^, *V*_total_ = 0.4772 cm^3^ g^−1^), was used as a support for NiMo-based deoxygenation catalysts. All catalysts were prepared so that they contained 2.6 wt.% Ni and 7.8 wt.% Mo. After impregnation, the materials were dried overnight at 120°C, followed by calcination in the air flow for 5 h at 450°C. NiO and MoO_3_ standards for XRD analysis were supplied by Sigma-Aldrich.

NiMo-alumina catalysts were prepared by impregnation of alumina support in ethanol or water solution. Ca. 100 ml of solvent was heated to 50°C (65°C for water), metal salts were dissolved and left to stir for 30 min, support was added, and solution was left to equilibrate for 1 h. When equilibrium was reached, temperature was raised to 65°C (85°C for water) and solvent was left to evaporate. Differences in the preparation were as follows:

NiMo-**OE** [**O**rganic precursors in **E**thanol: impregnation of alumina with ethanol solution of nickel acetate tetrahydrate ((CH_3_COO)_2_Ni.4H_2_O, Sigma-Aldrich)] followed by drying and calcination at 450°C for 5 h. This was followed by impregnation with molybdenyl acetylacetonate (C_10_H_14_MoO_6_, Acros Organics), drying, and calcination.

NiMo-**OW** [**O**rganic precursors in **W**ater: from water solution of nickel acetate tetrahydrate ((CH_3_COO)_2_Ni.4H_2_O, Sigma-Aldrich] and molybdenyl acetylacetonate (C_10_H_14_MoO_6_, Acros Organics). It has to be noted that while nickel acetate tetrahydrate is readily soluble in water, molybdenyl acetylacetonate is not, and during the preparation, it changed color from green to red (like iron rust).

NiMo-**IW** [**I**norganic precursors in **W**ater: from water solution of nickel nitrate hexahydrate (Ni(NO_3_)_2_.6H_2_O, Lachema) and ammonium heptamolybdate tetrahydrate ((NH_4_)_6_(Mo_7_O_24_).4H_2_O, Sigma-Aldrich)].

While N_2_ adsorption, XRD, XRF, UV-vis DRS, and H_2_-TPR results refer to the oxide catalyst precursors, SEM- and TEM-EDX, hydrodesulfurization and hydrodeoxygenation (HDO) analyses are valid for sulfide catalysts.

Concentration of the Ni, Mo, and Al species in the catalysts was determined by Elvatech desktop energy-dispersive XRF spectrometer Elva X equipped with a Ti anode X-ray tube.

X-ray diffractograms were recorded with Bruker AXE D8-Advance diffractometer using Cu Kα radiation with a secondary graphite monochromator.

UV-vis diffuse reflectance spectra (DRS) of granulated (0.25–0.50 mm diameter) catalysts were recorded using GBC CINTRA 303 spectrometer.

Specific surface areas of the catalysts were measured at the boiling point of the liquid nitrogen (77 K) on Carlo Erba Sorptomatic Model 1900.

Redox behavior and dispersion of NiMoO_x_ surface species were investigated by the temperature programmed reduction by hydrogen (H_2_-TPR) using the AutoChem 2920 (Micromeritics, USA). One hundred milligrams of sample in a quartz U-tube microreactor was oxidized in oxygen flow at 450°C for 60 min prior to the TPR measurement. The reduction was carried out from 50 to 1,050°C with a temperature gradient of 10°C/min in flow of reducing gas (5 vol.% of H_2_ in Ar, 25 ml/min). The changes in hydrogen concentration were monitored by the TCD detector. Calculation of oxidation state change is as follows:

$$\begin{array}{l} \Delta =\frac{2.n\left(H_{2}\right)}{\frac{m\left(metal\right)}{M_{r}\left(metal\right)}} \end{array}$$

where 2 is reduction reaction stoichiometry factor, *n*(H_2_) is number of moles of hydrogen gas [mol], *m*(metal) is mass of the metals present in the catalyst calculated from theoretical concentration of Ni and Mo [g], and *M*_r_(metal) is the molecular weight of the metals present in the catalyst [g mol^−1^].

Number of active sites in sulfided catalysts (temperature gradient of 5°C/min, at 400°C, 2 h under 50 ml min^−1^ of 10 vol.% H_2_S/H_2_, transferred under Ar atmosphere) was determined by oxygen chemisorption (Millman et al., 1984; Leglise et al., 1988; Hong and Regalbuto, 1995) using AutoChem 2920 (Micromeritics, USA). One hundred milligrams of sulfided sample in a quartz U-tube microreactor was flushed in helium flow at 300°C for 60 min prior to the chemisorption measurement. Oxygen from 2 wt.% O_2_ in He was let flow through sample at−78°C for 30 min. After that, the physisorbed oxygen was purged out by He flow and amount of chemisorbed oxygen was obtained by temperature-programmed desorption from −78 to 150°C with a temperature gradient of 20°C min^−1^ in flow of He (50 ml min^−1^). Oxygen uptakes were corrected by the oxygen uptake of alumina support.

Scanning electron microscopy (SEM-EDX) studies on sulfided catalysts (temperature gradient of 5°C/min, at 400°C, 2 h under 50 ml min^−1^ of 10 vol.% H_2_S/H_2_, transferred under Ar atmosphere) were performed using an HR-SEM Jeol 7600F microscope (operating at 30 kV) equipped with an X-ray energy-dispersive (EDX) microanalyzer (Oxford Instruments). EDX analyses of Ni, Mo, Al, S, and O were performed on multiple ca. 300 μm^2^ large areas (scan time of 75 s) of different grains to obtain representative statistical evaluation of the catalyst's composition.

High-resolution transmission electron microscopy (HR-TEM) studies on sulfided catalysts (temperature gradient of 5°C/min, at 400°C, 2 h under 50 ml min^−1^ of 10 vol.% H_2_S/H_2_, transferred under Ar atmosphere) were performed on a JEOL 2100F Field Emission Electron Microscope equipped with an ultra-high-resolution pole piece (coefficient of spherical aberration, Cs = 0.5 mm) operating at 200 kV. Finely ground solid samples were dispersed in ethanol solution and drops of supernatant liquid were deposited on holey carbon copper grids (300 mesh). EDX analyses of Ni, Mo, Al, S, and O were performed on multiple ca 430 nm^2^ large areas (scan time of 75 s) of different grains to obtain representative statistical evaluation of the catalyst's composition.

HDO catalytic tests were performed in a bench-scale catalytic apparatus equipped with an electrically heated fixed-bed reactor of 17 mm inner diameter. Prior to the experiment, pelleted and sieved catalyst (0.25–0.5 mm grain size) was divided into five equal amounts. These amounts were mixed with fine SiC of diameter 0.1 mm. Mixing was performed by volume, ranging from ratio 1:1 to 1:5 (catalyst:SiC) with the most diluted catalyst at the top where liquid feed enters the reactor. Before the loading of the prepared catalyst into the reactor, first, a small amount of the thermo-resistant wool was placed at the bottom, and onto it, 5 ml of coarse SiC (ø 1–2 mm) was introduced. The reactor was then filled with 1:1–1:5 mixtures, respectively. The next step included the loading of 10 ml of fine SiC, followed by adequate amount of coarse SiC to fill the reactor to the top. Food grade rapeseed oil was used as a liquid feed and refinery hydrogen gas (>99% purity) was used as a gas feed. The main impurities of hydrogen gas were methane (ca. 0.6%) and nitrogen (ca. 0.3%). Prior to the catalytic testing, catalysts mixed with silicon carbide were introduced into the reactor and sulfided. Sulfidation included several steps as follows. The catalyst in reactor was heated up to 200°C in hydrogen (5 MPa, 50 Nl h^−1^). Once the temperature of 200°C was reached, sulfiding agent (5 vol.% dimethyl disulfide in *iso*-octane) was introduced (1.17 ml min^−1^). The temperature gradient was set to 10°C h^−1^. After reaching 220°C, the slope was increased to 30°C h^−1^ until achieving the final temperature of 340°C, at which the catalyst was sulfided for the next 4 h. Reaction conditions used in the catalytic tests were as follows: *T* = 260°C; m(catalyst) = 5 g; p(H_2_) = 3.5 MPa; feed = food-grade rapeseed oil; H_2_:oil = 50 mol/mol; WHSV = 1–20 g(oil) g(catalyst)^−1^ h^−1^ [weight hourly space velocity, WHSV = 1/W:F (weight-to-mass flow ratio)].

Liquid deoxygenation products were analyzed after separation of water by gas chromatography. The analysis was performed with a Shimadzu GC-2010 gas chromatograph equipped with RESTEK MTX® Biodiesel TG film-coated column (*l* = 10 m, *d* = 0.32 mm, *t*_f_ = 0.1 μm) and flame ionization detector (FID). Conversion of rapeseed oil was calculated as:

$$\begin{array}{l} X=\frac{\left({c\left(\text{TG}\right)}_{0}-c\left(\text{TG}\right)\right)}{{c\left(\text{TG }\right)}_{0}}.100, \end{array}$$

where *c*(TG)_0_ and *c*(TG) are sums of the initial and final concentrations of triglycerides determined by the GC analysis (in %), respectively. Deoxygenation activity (oxygen elimination) was calculated as follows:

$$\begin{array}{l} \text{oxygen elimination}=\frac{\left({c\left(\text{oxygen}\right)}_{0}-c\left(\text{oxygen}\right)\right)}{{c\left(\text{oxygen}\right)}_{0}}\cdot 100 \end{array}$$

where *c*(oxygen)_0_ and *c*(oxygen) are sums of the initial and final concentrations of oxygen in reactants and products determined by the GC analysis (in %), respectively. Selectivity was calculated as follows:

$$\begin{array}{l} S_{i}=\frac{c_{i}}{\text{oxygen elimination}}\cdot 100, \end{array}$$

where *c*_*i*_ is concentration of the product (in wt.%) determined by GC analysis. Selectivity to oxygenates (*S*_oxo_) is the sum of the selectivities to all present fatty alcohols and acids. Selectivity to esters (*S*_ester_) is the sum of the selectivities to oleyl oleate and stearyl stearate. It should be noted that all catalysts in this study contain the same amounts of Ni and Mo, which enables a direct comparison of the catalysts in terms of their catalytic performance (oxygen elimination and selectivity values).

Hydrodesulfurization (HDS) catalytic tests were carried out in stainless steel reactors (Parr Instrument Company). The reaction of 4,6-DMDBT (4,6-dimethyldibenzothiophene) (150 mg dissolved in 100 ml of dodecane) was performed at 320°C, 5.52 MPa of H_2_ pressure, and 1,000 rpm. Sulfidation pretreatment in this case was carried out in the same way as for SEM and TEM analyses (see above). HDS products were analyzed by Varian CP-3800 GC equipped with an Agilent J&W HP-5 column and flame ionization detector. Initial reaction rates were calculated on the basis of pseudo-first-order approximation from conversion values. Conversion was calculated as follows:

$$\begin{array}{l} X=\frac{\left({c\left(DMDBT\right)}_{0}-c\left(DMDBT\right)\right)}{{c\left(DMDBT\right)}_{0}}.100 \end{array}$$

where *c*(DMDBT)_0_ and *c*(DMDBT) are sums of the initial and final concentrations of 4,6-DMDBT determined by the GC analysis (in %), respectively.

TOF (turnover frequency) values were calculated as follows:

$$\begin{array}{l} TOF=\\ \frac{n_{reactant}.X.N_{A}}{m_{cat}.n_{O_{2}}^{CHS}.N_{A}}\left[molecule{\left(number\;of\;active\;sites\right)}^{-1}\;s^{-1}\right] \end{array}$$

where *n*_reactant_ is the number of moles of reacting molecule [4,6-DMDBT [mol] or molar flow of rapeseed oil [mol s^−1^]], *X* is conversion (oxygen elimination), *N*_A_ is Avogadro's number (6.022 × 10^23^ mol^−1^), *m*_cat_ is the mass of the catalyst [g], and $n_{O_{2}}^{CHS}$ is the number of active sites expressed as an amount of chemisorbed oxygen [μmol g^−1^].

## Results

### Catalyst Characterization

Table 1 gives the values of the specific surface areas (*S*_BET_) for NiMo-OE, NiMo-OW, NiMo-IW, and the support. The impregnation of Ni and Mo onto the alumina support led to a decrease in *S*_BET_ in comparison with that of the fresh alumina support for all catalysts. NiMo-OE and NiMo-OW exhibit similar *S*_BET_ (257 and 244 m^2^ g^−1^, respectively) that are approximately twice as high as the value found for the NiMo-IW catalyst (137 m^2^ g^−1^). Practically no micropores are present in the materials and the porosity corresponds to the decreasing *S*_BET_ values (Table 1).

**Table 1:** Specific surface area and total pore volume of NiMo-alumina catalysts and change of oxidation state during their reduction.

**Sample**	**Note**	***S*_**BET**_, m^**2**^ g^**−1**^**	***V*_**total**_, cm^**3**^ g^**−1**^**	Δ, H_**2**_-TPR*
Alumina	Support	296	0.4772	-
NiMo-OE	Organic precursors, ethanol	257	0.3765	3.49
NiMo-OW	Organic precursors, water	244	0.3486	2.44
NiMo-IW	Inorganic precursors, water	137	0.2268	6.67

* *change of oxidation state calculated from the total hydrogen consumption*.

**Figure 1:**
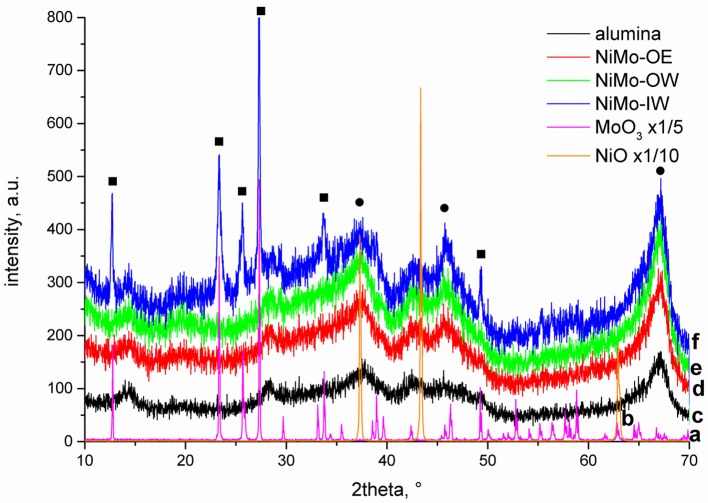
XRD diffractograms of the **(a)** MoO_3_, **(b)** NiO, **(c)** alumina support, **(d)** NiMo-OE, **(e)** NiMo-OW, and **(f)** NiMo-IW. Square denotes bulk MoO_3_ and circle γ-alumina phase, respectively.

The diffractograms of all investigated catalysts (Figure 1) exhibit maxima at 2θ angles (Miller indices in brackets) of 37.6 (311), 45.8 (400), and 67° (440) that can be attributed to the γ-alumina phase (JCPDS 10-0425). Their poor resolution points to the prevailing presence of an amorphous alumina phase. Diffraction lines characteristic to crystalline NiO are not evidenced. It is due to the low total amount of nickel (<3 wt.%) and its high dispersion. NiMo-IW contains crystalline MoO_3_ as confirmed by the visible MoO_3_ diffractions at 2θ angles of 12.74 (020), 23.36 (110), 25.64 (040), 27.34 (210), 33.7 (111), and 49.4° (002) (JCPDS 76-1003, 05-0508). NiMo-OW and NiMo-OE do not exhibit these reflections. Since the amount of Mo is the same in all three catalysts, it can be suggested that the impregnation by organic precursors (-OW and -OE) resulted in a better dispersed MoO_3_ phase or virtually exclusively amorphous phase.

**Figure 2:**
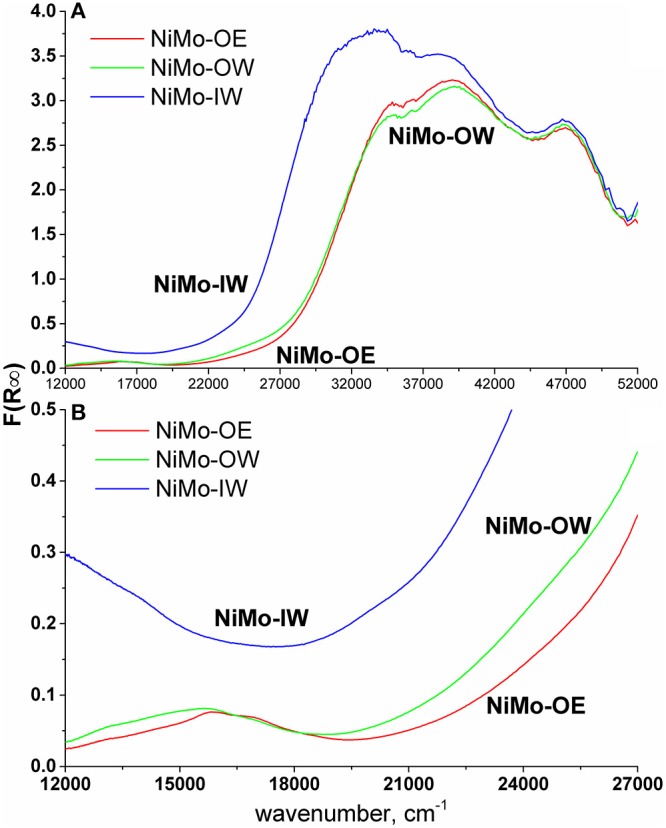
DR UV-vis spectra **(A)** and d–d region detail **(B)**.

DRS of NiMo catalysts shows a qualitatively similar trend (Figure 2A). The spectrum of NiMo-IW (when compared to NiMo-OW and NiMo-OE) displays red shift of the intense Mo-O CT (charge-transfer) band at 32,000 cm^−1^, which is indicative of bulk MoO_3_ formation as it was established by Weber (1995). He reported that the absorption edge energy (estimated from the plot of (F(R_∞_).E)^2^ vs. energy) decreases with the increasing Mo oxide domain size and he reported energy of 2.97 eV for bulk MoO_3_. The band gap energies are 3.29, 3.71, and 3.75 eV for NiMo-IW, NiMo-OW, and NiMo-OE, respectively, which indicates the highest proximity of NiMo-IW value to the bulk MoO_3_ value that is in line with the XRD results (Figure 1).

Elevated d–d region (visible range, ca. 12,000–28,000 cm^−1^) background (Figure 2B) in the spectrum of Ni-containing catalysts can be indicative of the presence of non-stoichiometric nickel oxide (Ni_2_O_3_) (Jitianu et al., 2000). This background is observed only in the spectrum of NiMo-IW.

The bands from ca 30,000 cm^−1^ to 52,000 cm^−1^ show non-specific Mo-O (Jeziorowski and Knoezinger, 1979; Plyuto et al., 1997; Guevara-Lara et al., 2007) and Ni-O charge-transfer transitions (Lepetit and Che, 1996; Capek et al., 2008; Priecel et al., 2011a). This region is mostly neglected because of the non-specificity and overlap of these transitions (Ni and Mo).

The bands below 30,000 cm^−1^ contain d–d transitions of Ni (Schoonheydt et al., 1987; Lepetit and Che, 1996; Zanjanchi and Ebrahimian, 2004; Nikolova et al., 2007; Priecel et al., 2011a) with a slight overlap of the red-shifted Mo-O CT band at 32,000 cm^−1^, the shoulder of which reaches as far as 25,000 cm^−1^ and could interfere with the interpretation of the Ni d–d transitions. At around 13,000–14,000 cm^−1^ and 24 000 cm^−1^, transitions of Ni(O_h_) occur (Lepetit and Che, 1996; Zanjanchi and Ebrahimian, 2004; Nikolova et al., 2007; Priecel et al., 2011a) and the region between 15,000 and 17,000 cm^−1^ is characteristic for the Ni(T_d_) bands (Schoonheydt et al., 1987; Lepetit and Che, 1996; Zanjanchi and Ebrahimian, 2004; Nikolova et al., 2007; Priecel et al., 2011a). It can be evidenced that NiMo-OW shows more intense Ni(O_h_) transitions at 13,000–14,000 cm^−1^ and a shoulder at 24,000 cm^−1^ when compared to NiMo-OE, which are indicative of a higher relative population of the Ni(O_h_) species in comparison with Ni(T_d_) species. In the case of NiMo-IW, this region is overlapped by aforementioned spectrum background (Figure 2).

**Figure 3:**
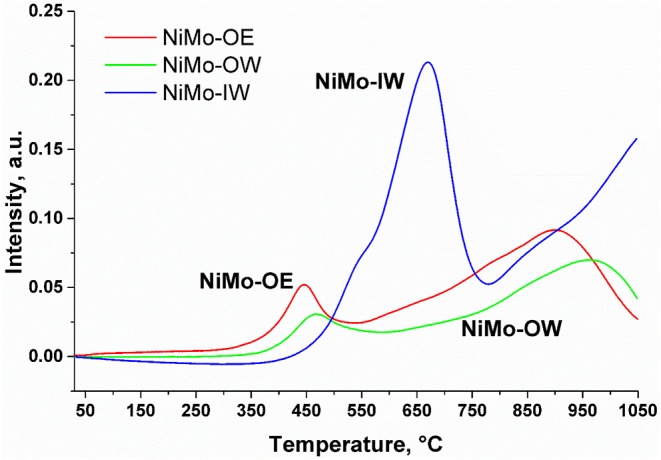
H_2_-TPR profiles of the catalysts NiMo-OE (red), NiMo-OW (green), and NiMo-IW (blue).

TPR-H_2_ profiles of NiMo-IW and NiMo-OW (Figure 3) show two distinct peaks with maxima in the regions of 350–550°C and 700–1,050°C. The first region is generally ascribed to the reduction of nickel oxides (Ni^2+^, Ni^3+^ → Ni^0^) (Zielinski, 1982; Rynkowski et al., 1993; Yang et al., 2009) and polymeric Mo(O_h_) species (Brito and Laine, 1993; Wang et al., 2008). The second one can be assigned to the reduction of mixed Mo(O_h_) and Mo(T_d_) species (Brito and Laine, 1993; Wang et al., 2008) and nickel spinel phase (NiAl_2_O_4_, NiAl_*x*_O_*y*_, where *x, y* are expected to be higher than 2 and 4, respectively) (Zielinski, 1982; Brito and Laine, 1993; Rynkowski et al., 1993; Yang et al., 2009). It is hard to distinguish the contribution of Ni and Mo species as the individual peaks overlap each other. In contrast to NiMo-OW, the NiMo-OE profile is slightly shifted to lower temperatures that reflects a better reducibility of Ni and Mo species in NiMo-OE. It is also associated with a higher change of the oxidation state observed for NiMo-OE (3.49) than for NiMo-OW (2.44). In contrast to NiMo-OW and NiMo-OE, the whole TPR profile of NiMo-IW is shifted to higher temperatures, so that the low-temperature region appears at 500–800°C, while the high-temperature region presents an unfinished reduction peak (800–1,050°C) whose maximum is most probably still hidden from view. It firstly indicates a harder reduction of Ni and Mo species present in NiMo-IW catalyst in comparison with the other two catalysts. The second feature worth noticing is the hydrogen consumption. When it is recalculated to the oxidation state change (Table 1), it is highest for NiMo-IW (Δ = 6.77). This implies that not only reduction of Ni [Δ = 2(3)] and Mo^6+^ → Mo^4+^ (Δ = 2) proceeds, but also to some extent Mo^4+^ → Mo^0^.

Number of active sites [μmol g^−1^] in sulfided catalysts determined by oxygen chemisorption follows the decreasing order NiMo-OW (23.1) > NiMo-OE (17.0) > NiMo-IW (14.9) (Table 3).

Supplementary Figure 1 displays SEM images and respective X-ray maps for Ni, Mo, Al, O, and S elements present in the sulfided catalysts. It can be seen that NiMo-OE and NiMo-OW (Supplementary Figures 1A,B) exhibit homogeneous distribution of all present elements and Ni, Mo, and S elements are present in the same areas as Al_2_O_3_ support, meaning even distribution of the active phase over the support. Mapping of NiMo-OW (Supplementary Figure 1B) also demonstrates uniform distribution of sulfide phase over the support, although the structure of the catalyst appears somehow different—more amorphous. Structure of NiMo-IW (Supplementary Figure 1C) is significantly distinct from those of NiMo-OE and NiMo-OW, showing large crystals (diameter up to 10 μm) containing high amount of Mo. It can be seen that these crystals are located in different positions than alumina phase and that they form a separate phase.

Table 2 shows concentration of Ni, Mo, Al, S, and O (S and O only applicable in SEM-EDX and TEM-EDX analyses of sulfided catalysts) as determined from XRF, SEM-EDX, and TEM-EDX. The Ni and Mo surface-reachable concentration is the highest in NiMo-OE followed by NiMo-OW. NiMo-IW shows very high surface Mo concentration (in Mo-rich areas up to 72 wt.% of Mo); however, the results also indicate great heterogeneity of the surface.

**Table 2:** Elemental composition (in wt.%) of NiMo-alumina catalysts as determined from XRF, SEM-EDX, and TEM-EDX.

**Technique**	**Element/catalyst**	**NiMo-OE**	**NiMo-OW**	**NiMo-IW**	
XRF	Ni	4.3	4.0	2.2	
	Mo	9.4	9.1	35.2	
	Al	21.3	21.5	11.8	
SEM-EDX	Ni	3.4	2.7	3.1	
	Mo	9.2	8.6	35.1	
	Al	36.2	34.8	16.0	
	O	43.9	46.5	32.1	
	S	7.3	7.4	13.7	
TEM-EDX (NiMo-IW 2 areas)	Ni	5.2	2.4	0.0	0.7
	Mo	12.9	8.0	15.3	72.3
	Al	64.8	44.8	51.5	1.6
	O	10.4	39.6	29.9	12.4
	S	6.7	5.2	3.4	13.0

The amount of remaining oxygen (not in the form Al_2_O_3_) and sulfur (not in MoS_2_) (in wt.%) could be calculated from the SEM-EDX (Table 2). For oxygen, an increasing trend can be found: NiMo-OE (26.7%) < NiMo-OW (33.4%) < NiMo-IW (55.6%) and for sulfur: NiMo-IW (0%) < NiMo-OE (15.7) < NiMo-OW (21.9). Moreover, the (Ni+Mo)-to-S ratio can be determined, and it is increasing in order: NiMo-OW (1.53) < NiMo-OE (1.72) < NiMo-IW (2.78). Note that for MoS_2_, the Mo-to-S ratio is 1.5. All these calculations indicate that NiMo-IW is not well-sulfided in comparison with NiMo-OE and –OW. However, the distinction between NiMo-OE and –OW in this sense is not so straightforward because of the similar values and, furthermore, the presence of the nickel.

**Figure 4:**
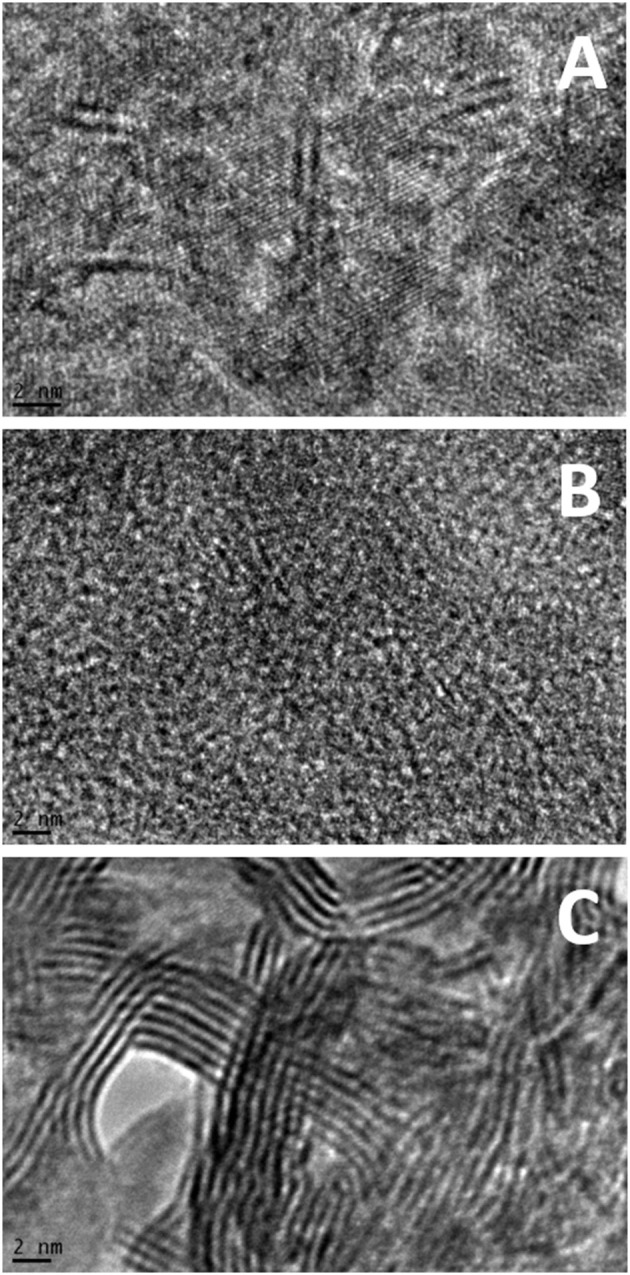
Example TEM images of sulfided NiMo-OE **(A)**, NiMo-OW **(B)**, and NiMo-IW **(C)** catalysts. Further images can be found in Supplementary Material.

Figure 4 and Supplementary Figure 2 show microstructure of the sulfided catalysts. Firstly, while NiMo-OE (Figure 4A) and NiMo-IW (Figure 4C) have readily visible structure of support phase, NiMo-OW (Figure 4B) has distinctive and amorphous structure with thicker sulfide phase particles. It is visible both from BF STEM (bright-field scanning transmission electron microscopy) (the darker the pattern, the heavier the element) (Supplementary Figures 2A,B) and from Z-contrast micrograph (opposite contrast to BF) (Supplementary Figures 2A,B). Secondly, Figure 4C shows the heterogeneity of NiMo-IW. The statistical evaluation of slab length and stacking was not possible, because except for the NiMo-IW bulk MoS_2_ phase (Figure 4C) (showing d-spacing of crystalline MoS_2_ phase), no d-spacing values corresponding to MoS_2_ could be found for NiMo-OE and NiMo-OW, even if the micrographs show structures that could correspond to the Mo or NiMo sulfide slabs.

### Catalytic Performance

**Figure 5:**
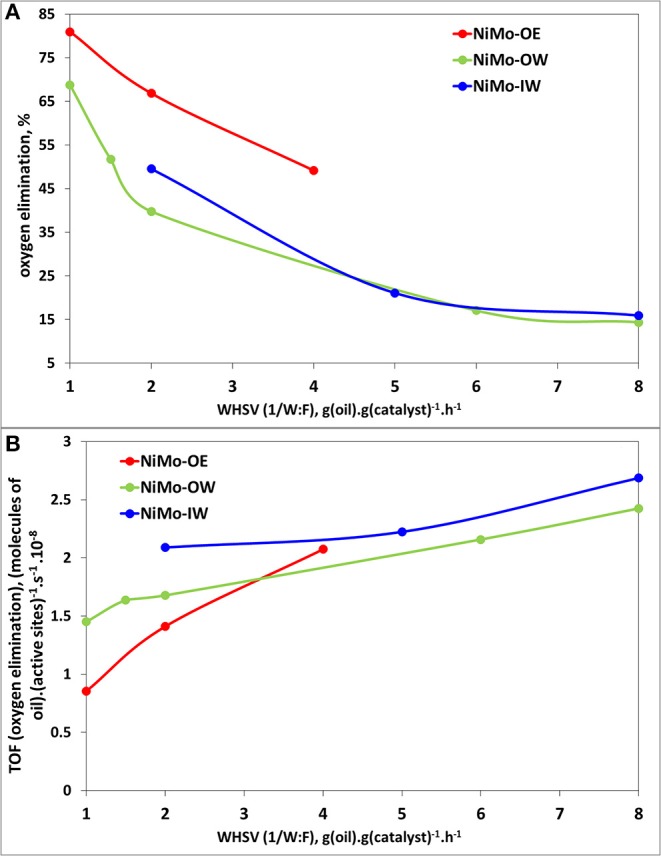
Catalytic activity plots for deoxygenation reaction: **(A)** Deoxygenation activity (oxygen elimination) vs. WHSV plot; **(B)** TOF (oxygen elimination) [active sites as determined from oxygen chemisorption (Table 3)] vs. WHSV plot. Reaction conditions: *T* = 260°C; *m*(catalyst) = 5 g; *p*(H_2_) = 3.5 MPa; feed = food-grade rapeseed oil; H_2_:oil = 50 mol/mol; WHSV = 1–8 g(oil) g(catalyst)^−1^ h^−1^.

Figure 5 shows the relation between oxygen elimination, i.e., how much of the initial oxygen atoms in triglyceride molecules (6) are eliminated by the HDO or hydrogenation to the final products (paraffins or alternatively olefins) and the WHSV (ratio of rapeseed oil mass flow to catalyst mass). Figure 5A demonstrates that NiMo-OE deoxygenates better than NiMo-OW and NiMo-IW, which exhibit a similar deoxygenation activity. On the other hand, when the oxygen elimination values are recalculated to TOF (Figure 5B) on the basis of chemisorbed oxygen, TOF oxygen elimination values could be viewed as very similar (with respect to experimental error).

With the increasing deoxygenation conversion, the concentration of oxygenates [reactants containing oxygen atom(s)] decreases and the concentration of hydrocarbons (alkanes) increases (not shown here for the sake of brevity; for more information, see Priecel et al., 2011a). Two main products are heptadecane and octadecane. The selectivities to heptadecane (Supplementary Figure 3) and octadecane (Supplementary Figure 4) increase, as expected, with the increasing oxygen elimination (deoxygenation conversion). NiMo-OW possesses slightly higher heptadecane and octadecane selectivities than NiMo-OE and NiMo-IW.

**Figure 6:**
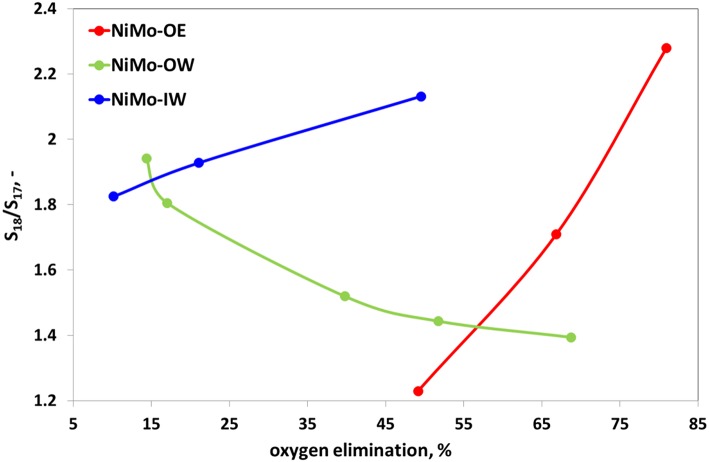
Catalytic activity plot for deoxygenation reaction, specifically ratio of selectivities to octadecane and heptadecane (S_18_/S_17_) vs. oxygen elimination. Reaction conditions: *T* = 260°C; *m*(catalyst) = 5 g; *p*(H_2_) = 3.5 MPa; feed = food-grade rapeseed oil; H_2_:oil = 50 mol/mol; WHSV = 1–20 g(oil) g(catalyst)^−1^ h^−1^.

The ratio between heptadecane and octadecane (Figure 6) reflects pathway preference between HDO and hydrodecarboxylation (HDC). While for NiMo-OE and NiMo-IW, this ratio increases with increasing deoxygenation conversion, the opposite trend can be identified for NiMo-OW. Table 3 gives the selectivities to main hydrocarbon products, oxygenates, and esters at the same conversion level.

**Table 3:** Amount of active sites (as determined from oxygen chemisorption), activities in HDS of 4,6-DMDBT [TOF initial reaction rate constants; (molecules of 4,6-DMDBT) (active sites in μmol g^−1^ as determined by oxygen chemisorption)^−1^ s^−1^] and selectivities in HDO of rapeseed oil (in %) at iso-conversion conditions [NiMo-OE (49.2%), NiMo-OW (51.7%), NiMo-IW (49.5%)].

**Catalyst**	**O_**2**_ adsorbed, μmol g^**−1**^**	**HDS TOF × 10^**9**^**	**HDO *S*_**17**_**	**HDO *S*_**18**_**	**HDO *S*_**oxo**_**	**HDO *S*_**ester**_**	**HDO *S*_**18**_/*S*_**17**_**
NiMo-OE	17.0	6.5	11.5	14.1	37.4	24.0	1.2
NiMo-OW	23.1	3.7	17.5	25.3	28.3	11.3	1.4
NiMo-IW	14.9	1.6	4.6	9.9	57.5	25.0	2.1

Supplementary Figure 5 shows selectivities to oxygenates (all present fatty alcohols and acids; without esters). It can be seen that selectivities to oxygenates over all catalysts follow one trend.

Selectivities to esters (both saturated and unsaturated) are depicted in Supplementary Figure 6. The shape of the *S*_ester_ curve is in agreement with the earlier suggested mechanism (Kubička, 2008) and can be explained by the following reasoning. Fatty esters are formed by the reaction of fatty acid and alcohol intermediates and can be deoxygenated to heptadecane and octadecane. Thus, a characteristic consecutive-reaction-intermediate curve, in which the concentration increases at first with the increasing conversion to reach a maximum and then decreases to some small constant value. In this case, the region around maximum can be seen. It can be observed that NiMo-OW is the least selective catalyst to esters, while the selectivity to ester is comparable for NiMo-OE and NiMo-IW.

## Discussion

### Deoxygenation Performance Enhancement

Firstly, there are several advantages associated with the use of organic precursors in ethanol (OE), but also in water (OW) in contrast to inorganic precursors in water (IW). Both NiMo-OE and NiMo-OW allow more facile transformation of the oxide form of catalyst to its active sulfided form, well-dispersed on the support (Supplementary Figure 1, Table 2). Elemental analysis (Table 2) supports that sulfidation of Ni and Mo is much higher in NiMo-OE and NiMo-OW than in NiMo-IW. In addition, it is clearly seen from SEM (Supplementary Figure 1) and TEM (Figure 4 and Supplementary Figure 2) measurements of sulfided catalysts that while NiMo-OE and NiMo-OW possess even distribution of active phase over the support, high phase heterogeneity is evidenced in the case of NiMo-IW. It is due to the larger fraction of MoO_3_ being present in the bulk form and thus not giving rise to the active NiMo sulfide phase, but rather to a bulk MoS_2_ phase, as detected in SEM (Supplementary Figure 1) and TEM (Figure 4 and Supplementary Figure 2) micrographs. Bulk MoS_2_ phase is intrinsically less active in deoxygenation than the NiMo phase as reported earlier (Kubička and Kaluža, 2010). Reduction of nickel (Ni^2+^ Ni^0^) is almost complete before the temperature of 1,000°C is reached, more so when the material is calcined at low temperature as 450°C (Smoláková et al., 2011). In addition, the presence of bulk MoO_3_ [as detected by XRD (Figure 1) and DRS (Figure 2)] promotes complete reduction of Mo (Mo^6+^ Mo^4+^ Mo^0^) to a higher degree (NiMo-IW) than when the molybdenum phase is well-dispersed (NiMo-OE and NiMo-OW). The formation of bulk MoO_3_ in NiMo-IW is connected with more pronounced decrease in specific surface area of NiMo-IW (137 m^2^ g^−1^) in comparison with NiMo-OE (257 m^2^ g^−1^) and NiMo-OW (244 m^2^ g^−1^). All of this explains the higher deoxygenation conversion (Figure 5) of NiMo-OE in comparison to NiMo-IW, but it does not explain the higher deoxygenation conversion of NiMo-OE in comparison to NiMo-OW.

The higher NiMo-OE deoxygenation conversion compared to NiMo-OW can hardly be explained by the higher specific surface area of this material (257 m^2^ g^−1^ NiMo-OE vs. 244 m^2^ g^−1^ NiMo-OW). In addition, both NiMo-OE and NiMo-OW catalysts are approximately the same with respect to the absence of crystalline NiO and MoO_3_ (XRD, Figure 1) and absence of bulk MoO_3_ (DRS, Figure 2). TEM study suggests that sulfide phase is more amorphous in the case of NiMo-OW when compared to NiMo-OE. The state of Ni can be discerned from UV-vis spectra in the visible region containing d–d transitions. Previously, we established that higher concentration of octahedral Ni species in the precursor (oxide form of Ni catalyst) of the active sulfide catalyst form promotes deoxygenation performance. However, this procedure is applicable only in the case of uniformity of all other catalyst parameters (Priecel et al., 2011a). In this case, it is clearly observed that the DR spectrum of NiMo-OW contains a higher population of Ni(O_h_) (transitions at 13,000–14,000 cm^−1^ and 24,000 cm^−1^) in comparison with NiMo-OE, which should evoke increased deoxygenation conversion. However, the state of molybdenum in NiMo-OE and NiMo-OW may not be the same considering the change of the Mo precursor mentioned in the experimental section (OW preparation; change of color of insoluble molybdenyl acetylacetonate at 65°C in the water from green to iron-rust red) and by the evaluation of TPR profiles (Figure 3) and TEM micrographs (Figure 4 and Supplementary Figure 2). The whole reduction profile of NiMo-OW (Figure 3) is shifted to higher temperatures and the change of oxidation state is 2.44 in comparison with 3.49 for NiMo-OE, which indicates higher reducibility of NiMo-OE than NiMo-OW, which subsequently leads to better level of sulfidation and oxygen elimination. Overall, this effect outweighs the contribution of Ni(O_h_) species, which, however, should also be mentioned. Despite the very high reducibility of the NiMo-IW (better than NiMo-OE and NiMo-OW), its deoxygenation conversion is lower due to the presence of bulk MoO_3_ phase (as discussed above). Since activation of the catalyst is carried out at 5.1 MPa of hydrogen and presence of the sulfiding agent, it is an indication that optimized reduction could play an indispensable role in the activation process. Furthermore, HDS tests were made to check the catalyst's performance in sulfur removal (Table 3). Initial reaction rate constants confirm that NiMo-OE is more effective in HDS than NiMo-IW and even NiMo-OW (6.5 × 10^−9^ vs. 3.7 × 10^−9^ [molecules of 4,6-DMDBT) (active sites)^−1^ s^−1^].

### Selectivities to Heptadecane and Octadecane

There are two major deoxygenation reaction pathways. HDO yielding hydrocarbons with even carbon atoms number and HDC yielding hydrocarbons with odd carbon atoms number when rapeseed oil (as a source of the mostly C_18_ fatty acid moieties bound in triglyceride molecule) is used as a feed. Sulfided Mo-alumina catalyst has been shown to deoxygenate rapeseed oil selectively by HDO, while sulfided Ni-alumina catalyst yielded almost exclusively HDC products (Kubička and Kaluža, 2010).

Concerning the two main deoxygenation pathways, decreasing selectivities order can be found to heptadecane and octadecane: NiMo-OW > NiMo-OE ~ NiMo-IW (Figures 5, 6). As high as >5% selectivity drop can be evidenced for NiMo-IW for both selectivities, in comparison with NiMo-OW. The drop in *S*_18_ could be explained by the formation of bulk MoO_3_, which cannot be transformed by sulfidation into well-dispersed and fully sulfided MoS_2_. The answer to the main difference in the state of nickel that could cause the *S*_17_ decrease for NiMo-IW can be found in the TPR profiles (Figure 3). The maximum of the first reduction process for NiMo-IW is located at ca. 100°C higher temperature than both NiMo-OE and NiMo-OW. It means that Ni is harder to reduce and to be sulfided in NiMo-IW catalyst. We can see from the surface elemental composition that amount of Ni in sulfided NiMo-IW catalyst is very low, which means that Ni is present probably deeper in the structure and cannot participate fully in the reaction and promote well the already badly dispersed MoS_2_. Moreover, it was reported that the main problem of good decoration of MoS_2_ edges by Ni (in the case of IW type of impregnation) is caused by the premature Ni sulfidation (takes place before sulfidation of Mo), which is caused by the higher tendency of Ni to undergo O-S exchange and that it can be improved using chelating agents (Medici and Prins, 1996). Furthermore, the drop of both *S*_17_ and *S*_18_ for NiMo-IW when compared to NiMo-OW could be caused by its higher preference for the esters formation from fatty acids and alcohols (*S*_ester_, Supplementary Figure 6). That way, when it comes to either reaction of acid, alcohol heptadecane, octadecane or acid + alcohol ester heptadecane, octadecane, the first reaction is more preferred for NiMo-OW, while the latter is more favored in the case of NiMo-IW.

The comparison of *S*_17_ and *S*_18_ (Supplementary Figures 3, 4) clearly shows that although NiMo-OE performs better than NiMo-OW in terms of overall oxygen elimination, the opposite situation can be noticed in this case. The increased selectivity of NiMo-OW to heptadecane could be explained by the increased population of octahedral Ni species when compared to tetrahedral Ni species, as it was already reported (Priecel et al., 2011a). Following the course of the *S*_18_ suggests also higher amount of HDO Mo sites. In this case the characterization offers only an ambiguous explanation. TPR profiles display the retarded reduction of both nickel and molybdenum; however, the exact position of the maxima for particular species can only be guessed at present. On the other hand, the increase in both *S*_17_ and *S*_18_ in the case of NiMo-OW with lower deoxygenation conversion in comparison with NiMo-OE could be further supported by the higher preference of NiMo-OE to form esters from fatty acids and alcohols when set against NiMo-OW. Also, it could be connected with higher amount of sulfur (in relation to amount of NiMo) present in the NiMo-OW than in NiMo-OE as determined from elemental composition of sulfided catalysts.

It was found in the previous studies that with increasing conversion (oxygen elimination), the *S*_18_/*S*_17_ ratio increased or was constant (Kubička and Horáček, 2011; Priecel et al., 2011a). The same conclusion could be applied to the *S*_18_/*S*_17_ ratio curve for NiMo-IW and NiMo-OE, although to different extents. These catalysts were prepared successfully and without problems, even though from different precursors and in various solvents (water and ethanol, respectively). However, an exactly opposite tendency can be observed for NiMo-OW catalyst. With decreasing content of oxygen in the products, the *S*_18_/*S*_17_ ratio decreases. This means that C_17_ formation increases with increasing conversion faster than that of C_18_. The aforementioned ratio decrease could be connected with the lower esters formation when compared to the NiMo-OE and NiMo-IW and with two obvious factors that could play role in the decrease of *S*_18_/*S*_17_ ratio, being change of Mo state during the impregnation (change of precursor color from green to iron-rust red) and its insolubility. The contrasting ratio of *S*_18_/*S*_17_ ratio for NiMo-OE and NiMo-IW was also found by Kaluža and Gulková (2016) [Ni(acac)_2_, MoO_2_(acac)_2_ in toluene vs. nickel nitrate and heptamolybdate] in HDS of 1-benzothiophene and hydrogenation (HYD) of 1-methyl-cyclohex-1-ene, where organometallic precursors gave catalysts of 13% higher HDS activity and lower HYD/DDS selectivities ratio (at 50% conversion). They suggested that HDS/DDS selectivities change almost independently of the preparation method and only due to the metals themselves.

## Conclusions

Three catalysts prepared from different precursors and in different solvents were studied. It was found that preparation of catalysts from organic precursors leads to well-dispersed sulfide phase and preservation of a high surface area, which facilitate improved deoxygenation activity when compared to a catalyst prepared from nickel nitrate and ammonium heptamolybdate. More specifically, the major conclusions reached are the following ones.

Impregnation of alumina with organic precursors (nickel acetate tetrahydrate and molybdenyl acetylacetonate) in ethanol (NiMo-OE) led to the catalyst, which offered better deoxygenation performance when compared to the conventional impregnation from nickel nitrate hexahydrate and ammonium heptamolybdate tetrahydrate in water (NiMo-IW). NiMo-OE displayed better preservation of high alumina support specific surface area (296 → 257 m^2^ g^−1^) in comparison with NiMo-IW (296 → 137 m^2^ g^−1^), better dispersed NiO and MoO_3_ phases on the support subsequently leading to the effective transformation of the oxide form of catalyst to its active sulfide form well-dispersed on the support. On the other hand, NiMo-IW catalyst contained high amount of bulk MoO_3_ (which translated to the formation of a separate bulk phases and lower extent of sulfidation) and exhibited distinct redox properties, which played a role in the catalyst pre-treatment and catalytic performance.

The same organic precursors used in impregnation in ethanol and water provided increase in deoxygenation activity for ethanol-prepared catalyst with respect to the water-prepared material (NiMo-OE vs. NiMo-OW, respectively). This was attributed to the worsened redox properties of NiMo-OW, probably caused by the change of Mo state during the catalyst preparation, which caused formation of well-dispersed but more amorphous and less active sulfide phase. It was also found that NiMo-OW catalyst demonstrated significantly lower preference to form esters than NiMo-IW and NiMo-OE.

Comparison of selectivities to heptadecane (*S*_17_) and octadecane (*S*_18_) revealed that NiMo-IW catalyst was the least selective to both hydrocarbons (the difference only minor in comparison with NiMo-OE, but significant with NiMo-OW). This low performance of NiMo-IW could be ascribed to the formation of bulk MoO_3_, which could not be transformed by sulfidation into well-dispersed MoS_2_, and also to the redox properties of the catalyst, which do not allow Ni in low surface concentration decorate well the already badly formed MoS_2_.

While for successfully carried out preparations (conventional and alternative impregnation—NiMo-IW and NiMo-OE, respectively), the ratio of selectivities to octadecane and heptadecane (*S*_18_/*S*_17_) increases with the increasing deoxygenation activity, the tendency is opposite (decreasing *S*_18_/*S*_17_ ratio with increasing activity) for NiMo-OW catalyst. It seems to be connected to the lower preference of NiMo-OW to form esters. In this case, two obvious factors could play a role, namely, the change of the Mo state during the impregnation (change of precursor color from green to iron-rust red) and its insolubility.

## Data Availability Statement

The datasets generated for this study are available on request to the corresponding author.

## Author Contributions

PP has synthesized the catalysts, performed and evaluated the catalytic tests, and took part in the manuscript preparation. DK has contributed to the experimental plan, helped in the experimental data analysis, and took part in the manuscript preparation. AV-Z and JA has analyzed the catalysts by TEM and contributed to the HDS experiments and their evaluation. MP has characterized the catalysts by XRF and analyzed the data. LČ has contributed to the experimental plan, helped in the characterization data analysis, and took part in the manuscript preparation.

### Conflict of Interest

PP was employed by the company Unipetrol Center for Research and Education. The remaining authors declare that the research was conducted in the absence of any commercial or financial relationships that could be construed as a potential conflict of interest.
