# Supplementary material for: Alternative Preparation of Improved NiMo-Alumina Deoxygenation Catalysts
Source: Front Chem. 2020 Apr 7;8:216. doi: 10.3389/fchem.2020.00216 (PMC7157445; doi:10.3389/fchem.2020.00216)
Supplement: Supplementary file 1 [file Data_Sheet_1.PDF]

Alternative preparation of improved NiMo-alumina deoxygenation catalysts

Peter Priecel[1,2], David Kubička[3,*], Armando Vázquez-Zavala[4], José Antonio de los Reyes[4], Miroslav Pouzar[1], Libor Čapek[1,*]

[1] Department of Physical Chemistry, Faculty of Chemical Technology, University of Pardubice, Studentská 573, 532 10 Pardubice, Czech Republic

[2]Unipetrol Centre for Research and Education, Záluží 1, 436 70 Litvínov, Czech Republic

[3]Department of Petroleum Technology and Alternative Fuels, University of Chemistry and Technology Prague, Technická 5, 166 28 Prague, Czech Republic

[4]Departamento de Ingeniería de Procesos e Hidraulica, Universidad Autónoma Metropolitana-Iztapalapa, San Rafael Atlixco No. 186, C.P. 09340, México, D.F., México

*** Correspondence:**
Libor Čapek and David Kubička
libor.capek@upce.cz and david.kubicka@vscht.cz

Supplementary Material

1 Supplementary Figures

A

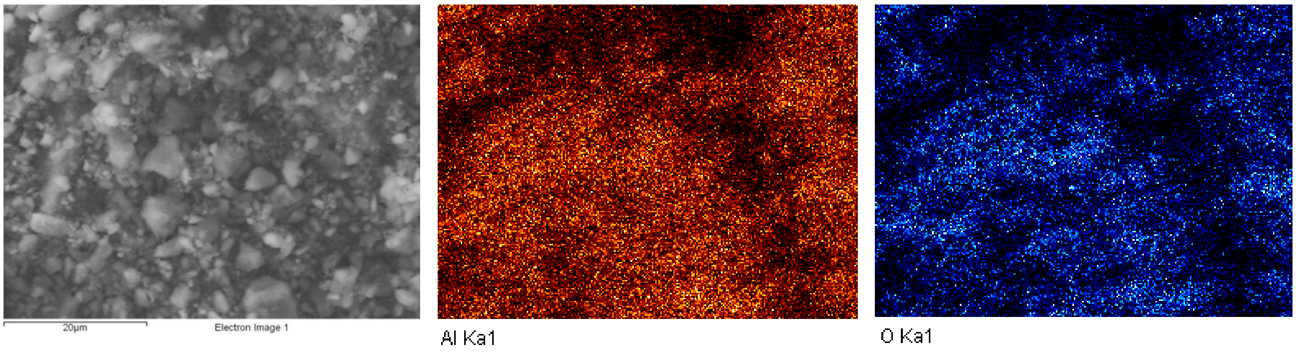

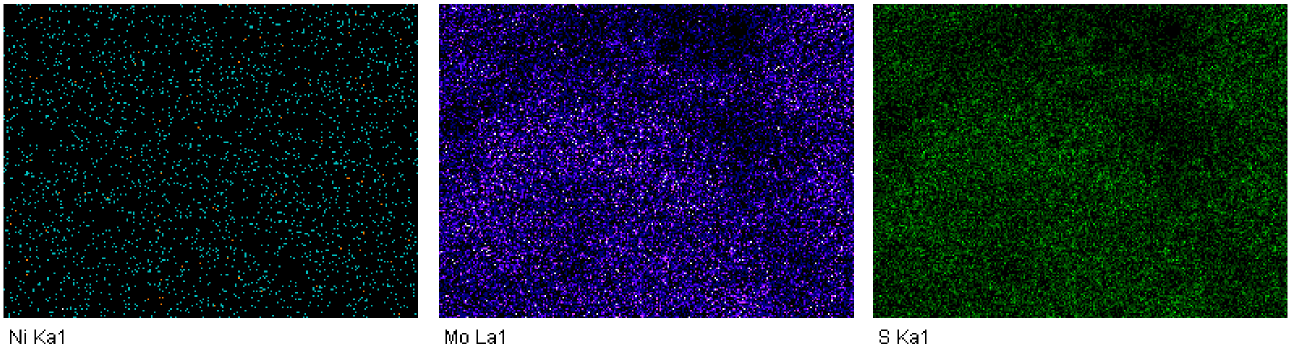
Ni Ka1
Mo La1
S Ka1

B

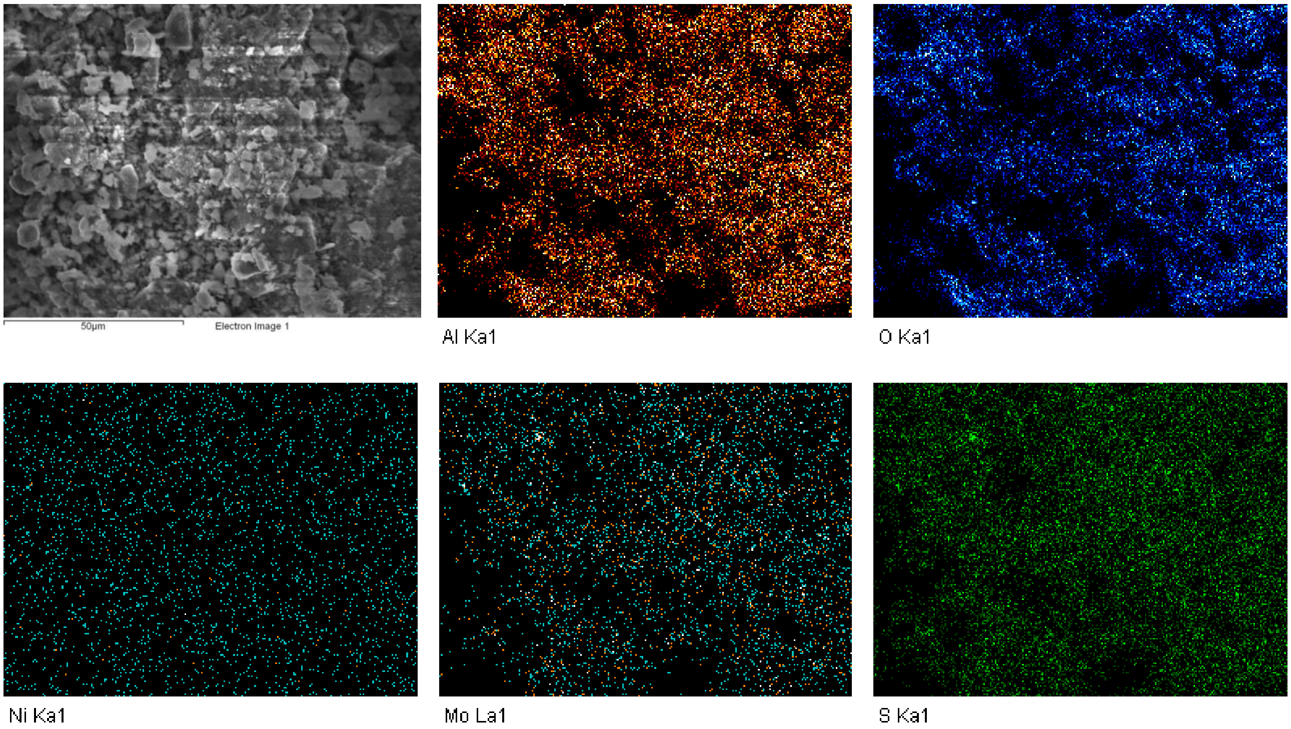
50µm
Electron Image 1
Al Ka1
O Ka1
Ni Ka1
Mo La1
S Ka1

C

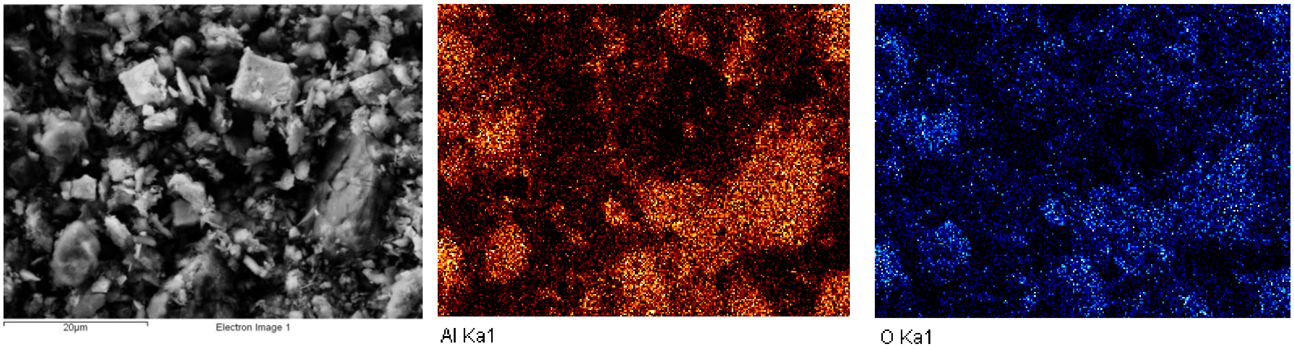
20µm
Electron Image 1
Al Ka1
O Ka1

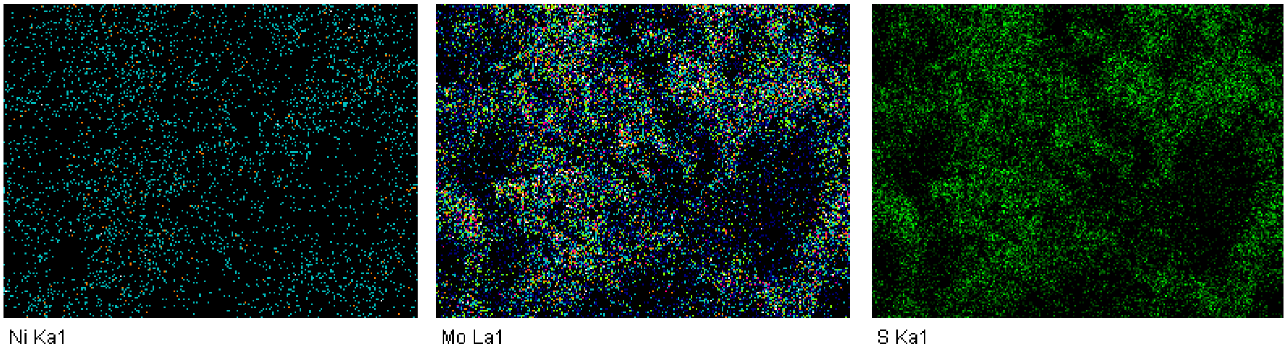

Supplementary Figure 1. SEM and SEM-EDX micrographs of sulfided A) NiMo-OE, B) NiMo-OW, C) NiMo-IW catalysts. X-ray maps are taken from the same area as representative SEM micrograph at the same magnification.

A

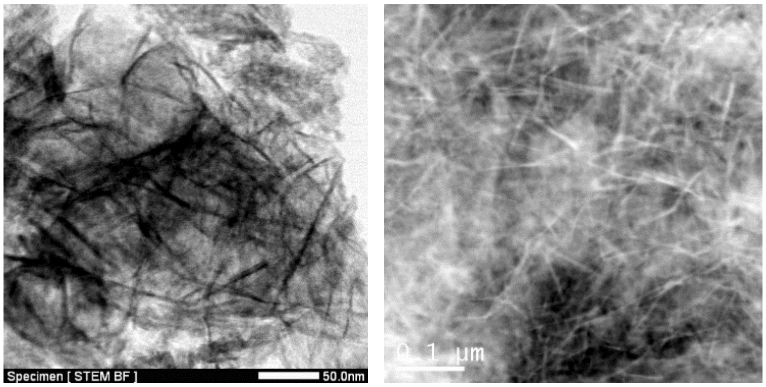

B

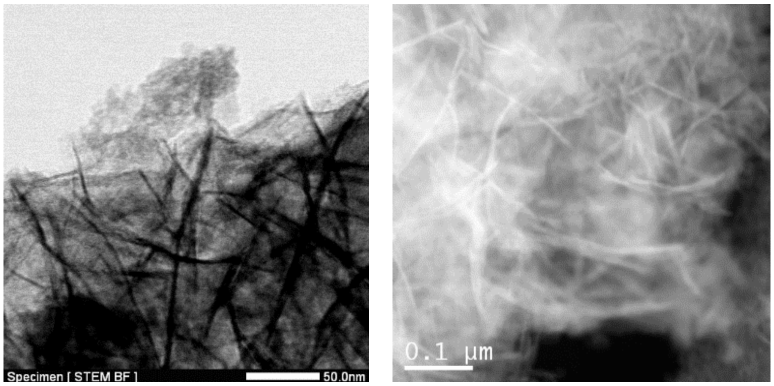

C

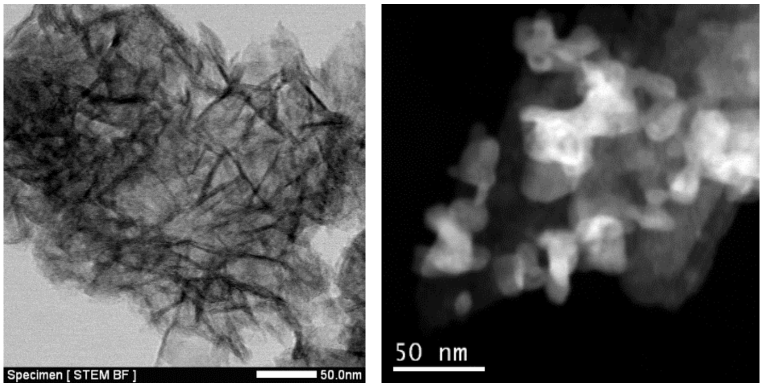

Supplementary Figure 2. STEM BF (left; bright field scanning transmission electron microscopy) and Z-contrast micrographs (right) of sulfided A) NiMo-OE, B) NiMo-OW, C) NiMo-IW catalysts.

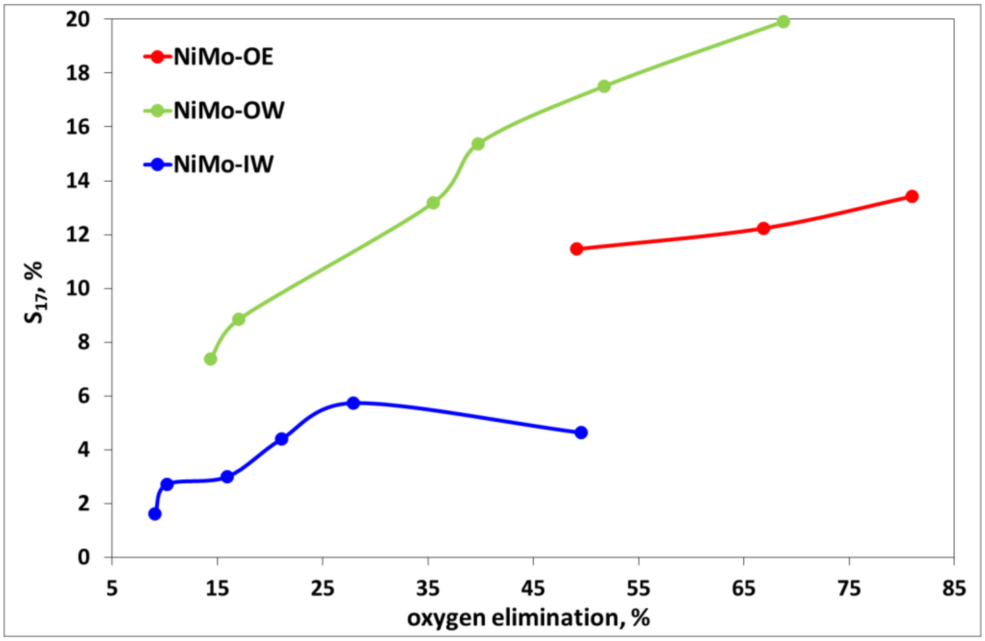

Supplementary Figure 3. Selectivity to heptadecane, S_{17} vs. oxygen elimination. Reaction conditions: T = 260 °C; m(catalyst) = 5 g; p(H_2) = 3.5 MPa; feed = food-grade rapeseed oil; H_2:oil = 50 mol/mol; WHSV = 1 – 20 g(oil).g(catalyst)$^{-1}$.h^{-1}.

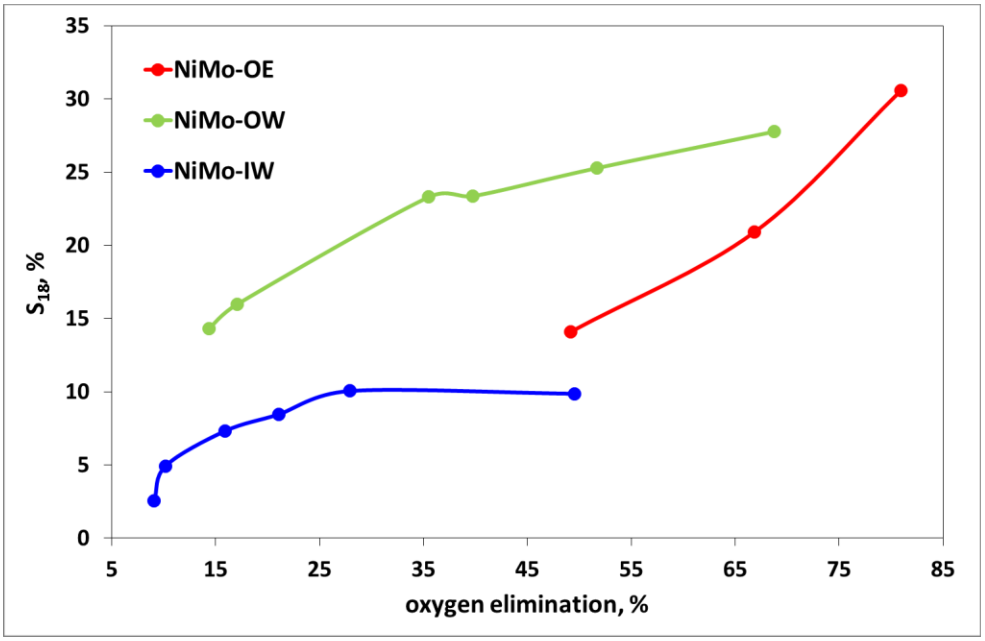

Supplementary Figure 4. Selectivity to octadecane, S_{18} vs. oxygen elimination. Reaction conditions: T = 260 °C; m(catalyst) = 5 g; p(H_2) = 3.5 MPa; feed = food-grade rapeseed oil; H_2:oil = 50 mol/mol; WHSV = 1 – 20 g(oil).g(catalyst)$^{-1}$.h^{-1}.

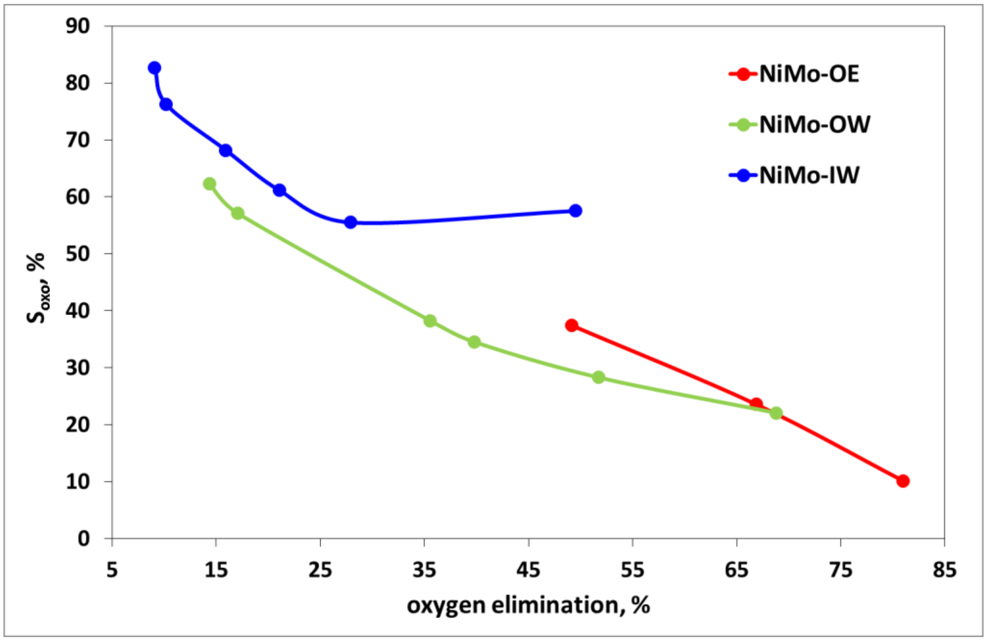

Supplementary Figure 5. Selectivity to oxygenates, S_{oxo} vs. oxygen elimination. Reaction conditions: T = 260 °C; m(catalyst) = 5 g; p(H_2) = 3.5 MPa; feed = food-grade rapeseed oil; H_2:oil = 50 mol/mol; WHSV = 1 – 20 g(oil).g(catalyst)$^{-1}$.h^{-1}.

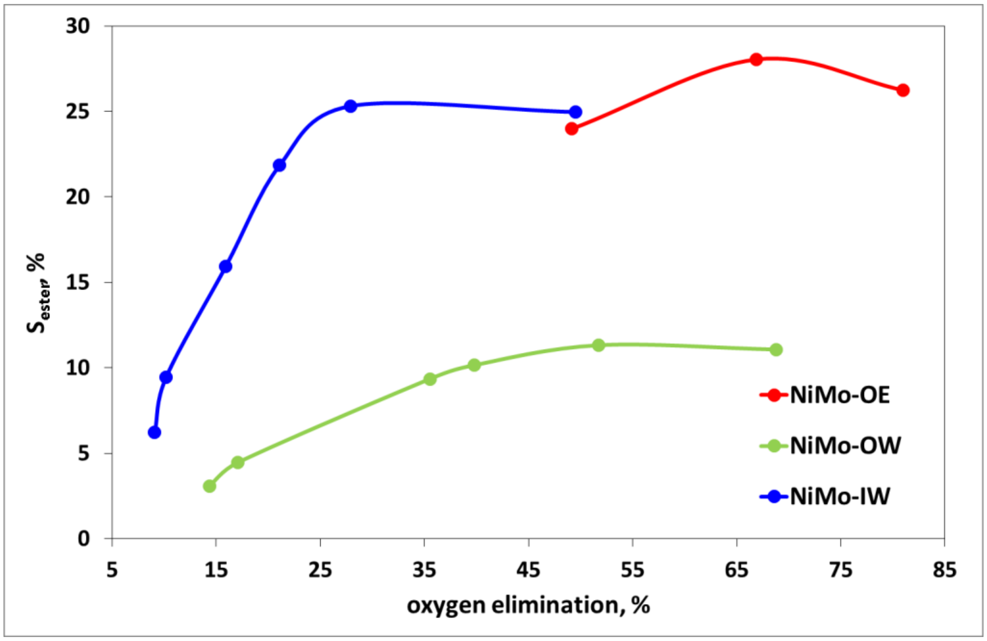

Supplementary Figure 6. Selectivity to esters, S_{ester} vs. oxygen elimination. Reaction conditions: T = 260 °C; m(catalyst) = 5 g; $p(H_2)$ = 3.5 MPa; feed = food-grade rapeseed oil; H_2:oil = 50 mol/mol; WHSV = 1 – 20 g(oil).g(catalyst)$^{-1}$.h^{-1}.
